# Editorial: IgA and mucosal immunity in vaccinology and in protection from infection

**DOI:** 10.3389/fcimb.2024.1409111

**Published:** 2024-04-10

**Authors:** Rita Carsetti, Isabella Quinti

**Affiliations:** ^1^ B Cell Pathophysiology Unit, Immunology Research Area, Bambino Gesù Children’s Hospital IRCCS, Rome, Italy; ^2^ Department of Molecular Medicine, Sapienza University of Rome, Rome, Italy

**Keywords:** IgA, mucosal immunity, immunization, protection, milk

Specific and neutralizing antibody titers are considered the best correlates of protection after vaccination or infection. The concentration of serum antibodies of both IgG and IgA isotypes, however, does not reflect mucosal immunity. IgA is the most abundant antibody at mucosal sites and can be detected in various biological specimens, including saliva, tears, and breast milk. The Research Topic aims to better define the IgA response to immunization in the serum and at mucosal sites. Infection and/or vaccination induce the production of protective antibodies of IgG isotype in the serum. These antibodies have a high affinity for the stimulating antigen and bind and neutralize pathogens, but do not prevent infection at mucosal sites.

Several vaccines against SARS-CoV-2 have been approved for use all over the world. Whereas in Western countries, mRNA and adenovirus-based products were administered to the large majority of the population, the inactivated vaccine BBIBP-CorV was widely used in China. Similar to all other vaccines, also two doses of BBIBP-CorV induced a strong immune response which declined three months later. A booster dose increased antibody levels and affinity (Wu et al.). Although all vaccines, including BBIBP-CorV, protect from hospitalization and severe disease, infection and reinfection remain unstoppable. The question of whether systemically administered vaccines can guarantee protection at the site of viral entry and induce IgA production is still open.

There is limited knowledge on the affinity, neutralizing activity, and longevity of mucosal IgA. We cannot measure to what extent vaccines administered intramuscularly contribute to immune protection at mucosal sites. These questions have been prioritized by the COVID-19 experience that has demonstrated the inability of effective vaccines and also hybrid immunity to prevent infection.

Two papers in this Research Topic address the role of mucosal immunity to SARS-Cov-2.

Stolovich-Ran et al. show that after intramuscular administration of mRNA vaccines, high levels of neutralizing IgG antibodies can be detected in the serum, whereas the increase of IgA is variable and transient. Most importantly, vaccine-induced IgA is monomeric and devoid of neutralizing ability. In contrast, IgA in the saliva, although produced in quantities that are equally variable and transient, is of the secretory type, i.e. polymeric, and can neutralize the virus. Injectable vaccines administered to naïve individuals did not induce significant mucosal IgA responses, but vaccination was able to boost pre-existing mucosal immunity thus underlining the importance of vaccination following natural infection.

Conti et al. studied the salivary IgA response to mRNA vaccination of children by separately measuring IgA1 and IgA2 levels. IgA1 is the most abundant isotype in the serum, whereas IgA2 is more represented at mucosal sites and is more often dimeric. Infected children had significantly higher levels of salivary RBD-specific IgA2 compared to IgA1, indicating that infection induces a specific mucosal immune response in children. Vaccination influenced the levels of RBD-specific IgA1, but had no effects on IgA2 (REF DOBBIAMO CHIEDERE).

These results indicate that serum antibodies partially contribute to mucosal protection, but locally produced polymeric IgA plays a most important role suggesting that mucosa vaccination may be a useful tool for preventive strategies.

The relative independence of mucosal and systemic immunity is also demonstrated in the case of influenza vaccination. Influenza vaccination of pregnant women is recommended to prevent infection of the mother and, at the same time, protect the child in the first months of life. Maternal antibodies of IgG isotype are transferred to the child through the placenta in the last trimester of pregnancy. After birth, the mother contributes to the immune protection of the child through breastfeeding. Yang et al. investigated whether IgA antibodies in maternal milk increase after seasonal influenza vaccination. They found a modest effect of maternal vaccination on the concentration and neutralization capacity of IgA in breastmilk. These results suggest that novel vaccines and vaccination strategies should be designed to increase antibody levels in the maternal milk thus improving its protective potential.

RSV is a respiratory virus causing severe disease at extreme ages, in infants and the elderly. Mucosal vaccines may represent the ideal choice to generate local immunity. An adenoviral vaccine encoding for the RSV fusion protein F, alone or in combination with IL1beta, was administered to mice (Maier et al.). The Authors report that while vaccination with Ad-F alone was immunogenic, the inclusion of Ad-IL-1β increased the levels of F-specific mucosal IgA and the frequency of tissue-resident memory T cells (TRM). Vaccine-induced immunity exerted more effective protection against RSV infection than natural immunity generated by a previous infection.

Neonates and infants have an increased susceptibility to pneumococcal infections. Because of the immaturity of the immune system, the response to vaccination is poor in the first months of life. The role of adjuvants in improving neonatal immunity was studied in a murine model. The pneumococcal conjugate vaccine Pn1-CRM197 was administered with the adjuvants dmLT or mmCT via the subcutaneous or intranasal route. Both adjuvants enhanced the level and persistence of the neonatal immune response following mucosal or parenteral immunization indicating that dmLT and mmCT are promising adjuvants for early-life vaccination strategies (Estupiñan et al.).

Several pathogens have developed mechanisms to inactivate IgA by proteolytic digestion of the hinge region. Rabbits have 15 IgA subclasses with exclusive hinge region motifs of varying lengths. de Sousa Pereira et al. swapped the human IgA hinge region with those of rabbit IgA. Thanks to this interesting strategy, they constructed a chimeric IgA1 able to resist bacterial proteolysis. The alternate hinge region did not affect antigen binding or Fca-mediated effector function. Proteolysis-resistant IgA may be used as a therapeutic antibody for mucosal administration.

The different papers included in this Research Topic increase our knowledge of IgA and its role in the upper respiratory tract. Specific and neutralizing antibody titers are considered the best correlates of protection after vaccination. The concentration of serum antibodies of both IgG and IgA isotype, however, does not reflect mucosal protection. Although antibodies contained in the serum reach mucosal sites by transudation, local immunity exerts a more effective defense through the function of resident memory B and T cells and the production of dimeric secretory IgA. Further research is necessary to dissect the mechanisms of immunity in the respiratory tract. Although IgA antibodies are detected in various biological specimens, including serum, saliva, and breast milk, they may have different origins and mechanisms of induction. Further investigation is necessary on the cellular types and the reactive sites responsible for IgA production. With this knowledge, we will be able to design mucosal vaccines for protective and durable local immunity.

## Author contributions

IQ: Writing – original draft, Writing – review & editing. RC: Writing – original draft, Writing – review & editing.

